# Supplementary material for: HLA-DRB1 Alleles Associated with Lower Leishmaniasis Susceptibility Share Common Amino Acid Polymorphisms and Epitope Binding Repertoires
Source: Vaccines (Basel). 2021 Mar 17;9(3):270. doi: 10.3390/vaccines9030270 (PMC8002611; doi:10.3390/vaccines9030270)
Supplement: Supplementary file 1 [file vaccines-09-00270-s001.pdf]

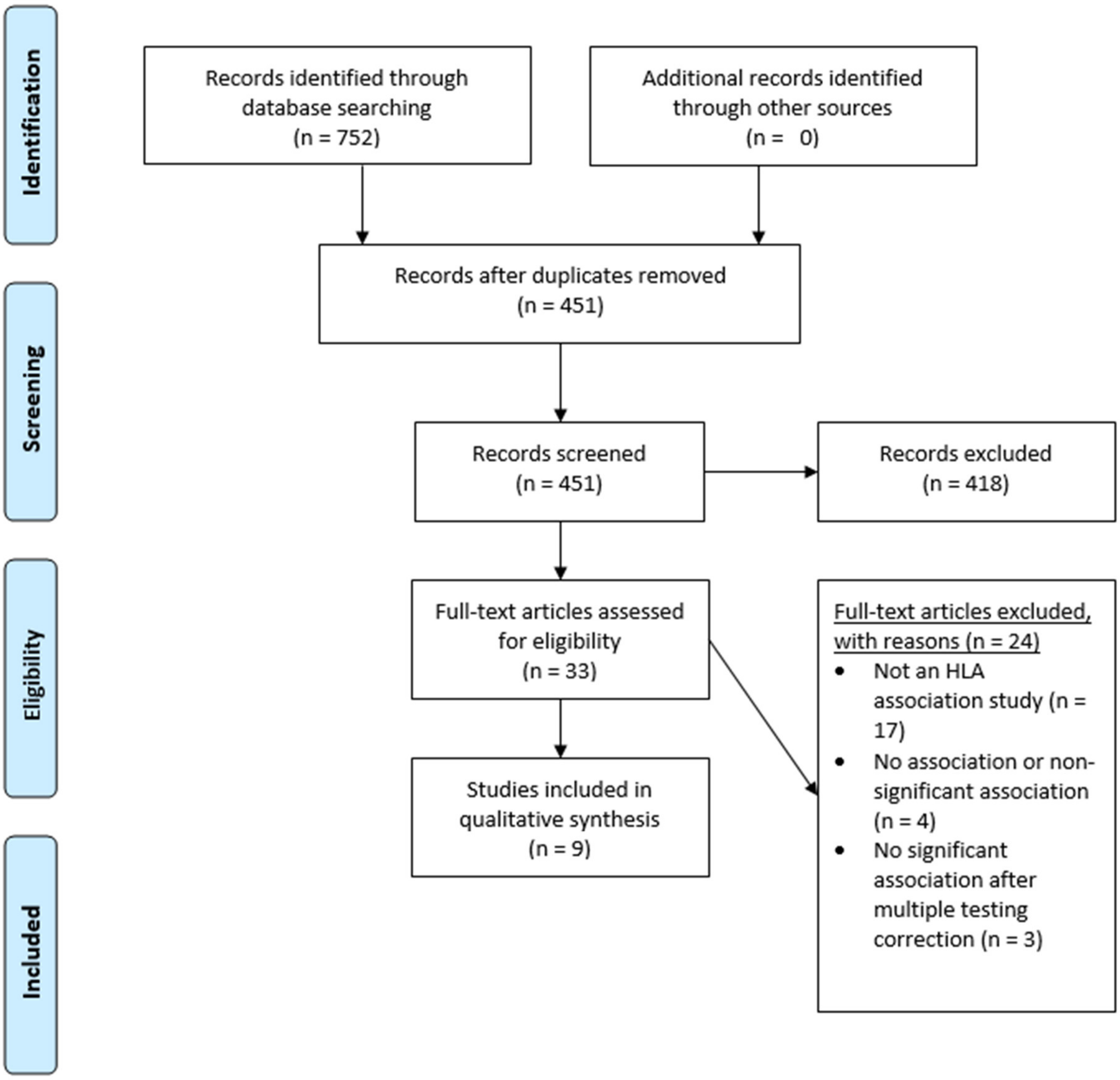

Figure S1. The PRISMA Flow Diagram used in this study to describe the literature review workflow [22].

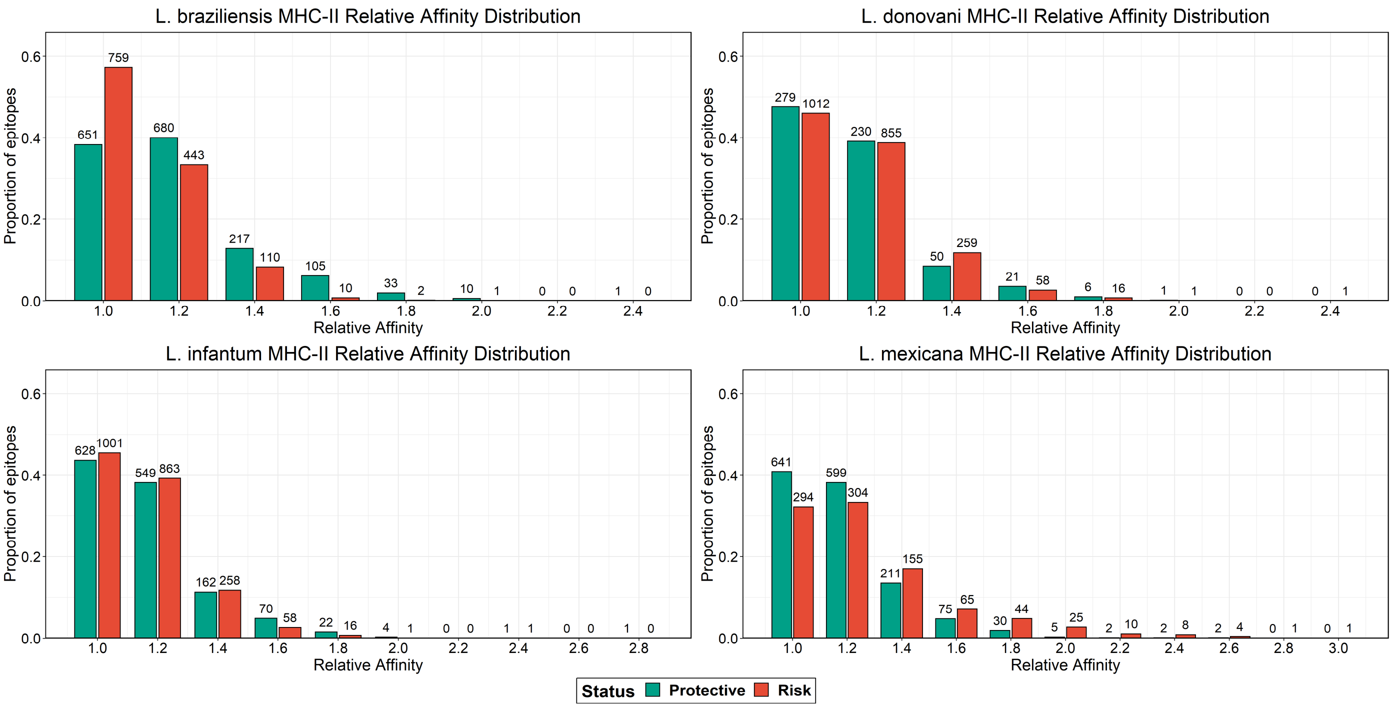

Figure S2. Relative affinity distribution plots for the HLA class II alleles associated with *L. braziliensis*, *L. donovani*, *L. infantum* and *L. mexicana*. This plot shows the number of epitopes, and the proportion of epitopes relative to the total number of epitopes, with a specific relative affinity.

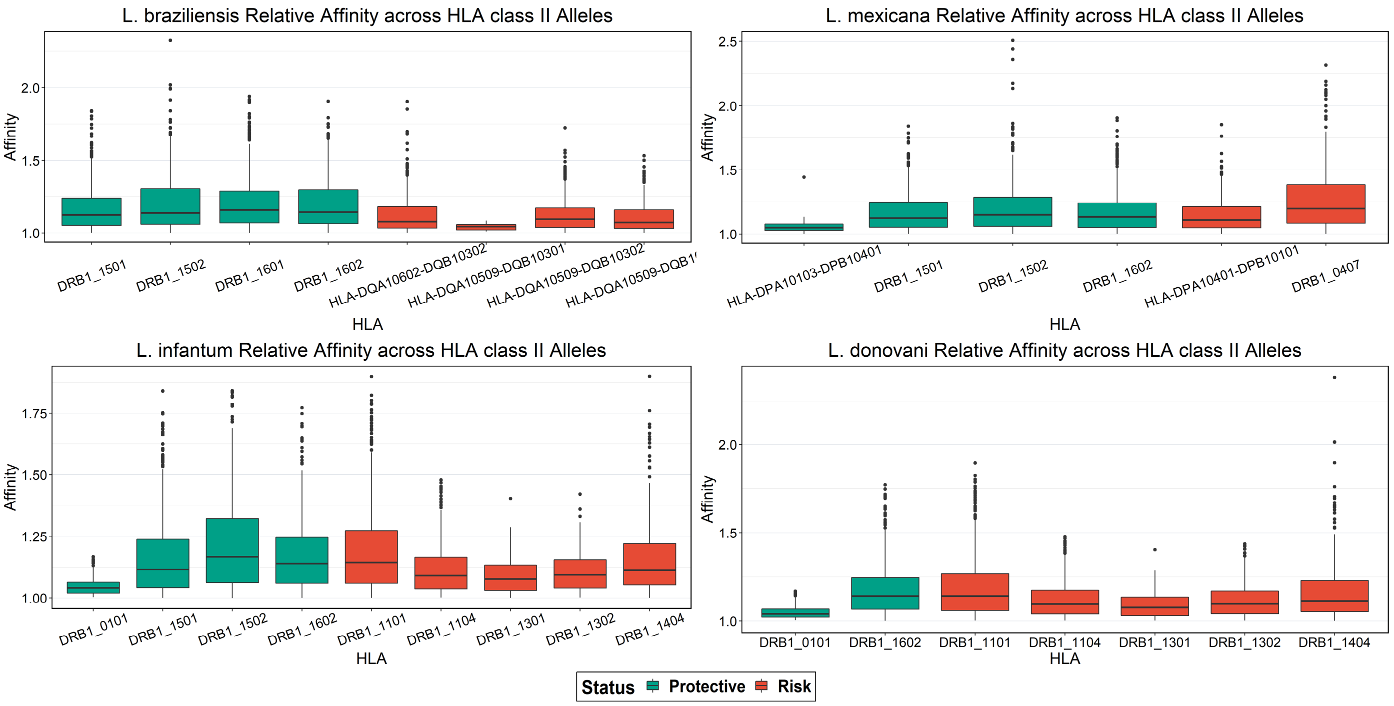

Figure S3. Relative affinity distribution across HLA class II alleles associated with protection and risk for *L. braziliensis*, *L. donovani*, *L. infantum* and *L. mexicana*.
